# Severe Disfiguring Scalp and Facial Oedema due to Henoch–Schönlein Purpura in a Child

**DOI:** 10.1155/2020/8823611

**Published:** 2020-09-14

**Authors:** Visvalingam Arunath, Arjuna Salinda Athapathu, Thabitha Jebaseeli Hoole, Heshan Aruppala, Asanka Rathnasri, Randula Ranawaka, Sachith Mettananda

**Affiliations:** ^1^University Paediatrics Unit, Colombo North Teaching Hospital, Ragama, Sri Lanka; ^2^University Paediatrics Unit, Lady Ridgeway Hospital, Colombo, Sri Lanka; ^3^Department of Paediatrics, Faculty of Medicine, University of Colombo, Colombo, Sri Lanka; ^4^Department of Paediatrics, Faculty of Medicine, University of Kelaniya, Ragama, Sri Lanka

## Abstract

Henoch–Schönlein purpura is a small vessel vasculitis that usually presents with palpable purpura, arthritis, abdominal pain, and nephritis. Subcutaneous oedema of dependent areas is common; however, oedema in the scalp is extremely rare especially in children older than two years. Here, we report a child with massive disfiguring scalp and facial oedema due to Henoch–Schönlein purpura. An eight-year-old boy presented with characteristic palpable purpuric rash and extensive disfiguring scalp and facial swelling for five days. He complained of blurred vision, vomiting, and severe headache on the day of admission. Examination revealed an ill child with extensive oedema of the face and scalp that was tender on palpation. His blood pressure was above the 99^th^ percentile, and he had exaggerated deep tendon reflexes and extensor plantar responses. All biochemical investigations including renal function tests were normal. Noncontrast CT head showed normal brain, with marked soft tissue swelling of the scalp. Ultrasonography showed soft tissue oedema within and surrounding facial muscles without evidence of neck vessel compression. Urine analysis revealed microscopic haematuria on day 14 of the illness, and immunohistochemical staining of renal biopsy confirmed Henoch–Schönlein purpura nephritis. In conclusion, this case report presents a child with severe, disfiguring scalp and facial oedema due to Henoch–Schönlein purpura. It highlights that severe subcutaneous oedema of Henoch–Schönlein purpura can involve any part of the body not limiting to dependent areas.

## 1. Introduction

Henoch–Schönlein purpura (HSP) is a small vessel vasculitis which is characterized by palpable purpura, arthritis, abdominal pain, and nephritis [1]. Although subcutaneous oedema of dependent areas has often been reported, scalp oedema in older children with HSP is extremely rare. Here, we report an 8-year-old boy with massive and disfiguring scalp and facial oedema due to HSP.

## 2. Case Report

An 8-year-old Sri Lankan boy presented with an erythematous rash, abdominal and lower limb pain, and gradually worsening swelling of the face and scalp for 5-day duration. He complained of blurring of vision, vomiting, and severe headache on the day of admission. The abdominal pain was episodic, and he had normal bowel habits with no history of haematemesis, melena, or blood-stained stools. There were no seizures, haematuria, frothy urine, or other urinary symptoms. He has had an upper respiratory tract infection one week before the admission.

**Figure 1:**
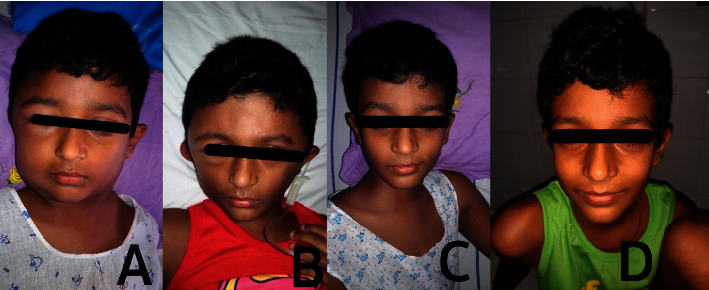
A series of photographs of the child demonstrating severe, disfiguring scalp and facial oedema and its gradual resolution.

**Figure 2:**
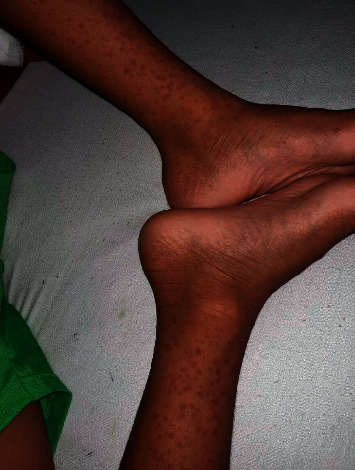
A photograph demonstrating classical nonblanching and a palpable purpuric rash over extensor surface of legs.

On examination, the child had severe and disfiguring nonpitting oedema of the scalp and face which was tender on palpation (Figure 1(a)). He had a palpable purpuric rash over the extensor surfaces of both lower limbs (Figure 2) and arthritis of the bilateral knee and ankle joints with restricted movements. His pulse and respiratory rates were 100 beats/minute and 20 breaths/minute, respectively. His blood pressure was 136/92 mmHg which was above the 99^th^ centile for the age and sex. The abdomen was soft and nontender without palpable masses or organomegaly. Nervous system examination revealed a conscious and well-oriented child with a Glasgow Coma Scale of 15/15. He had normal muscle tone and power in all four limbs with exaggerated deep tendon reflexes and extensor plantar response in bilateral lower limbs. His pupils, fundi, and cranial nerves were normal, and he did not have neck stiffness or Kernig's sign.

**Figure 3:**
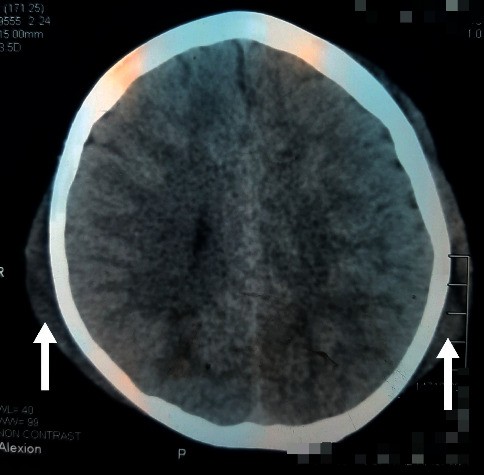
Noncontrast CT scan showing the soft tissue swelling of the scalp (arrows).

His complete blood count revealed haemoglobin: 14.0 g/dl, white cell count: 8.9 × 10^9^/l (neutrophils: 72% and lymphocytes: 26%), and platelet count: 498 × 10^9^/l. His blood film, C-reactive protein, erythrocyte sedimentation rate, serum electrolytes, hepatic transaminases, and serum albumin were normal. On admission, his urine full report did not show any abnormalities, and urine protein creatinine ratio, serum creatinine, and blood urea nitrogen were also normal. The complement C3 level was 113 mg/dl (normal 83–177), C4 level was 21 mg/dl (normal 12–36), and antistreptolysin O titre was 106 IU/ml (normal <200). Noncontrast CT scan of the head showed normal brain with no evidence of cerebral oedema with a marked soft tissue swelling of the scalp (Figure 3). Ultrasonography of the neck and face showed soft tissue oedema within and surrounding facial muscles without evidence of neck vessel compression. Abdominal ultrasonography, renal Doppler studies, electrocardiogram, and echocardiogram were normal. MRI of the brain was not performed due to limitation in resources.

The diagnosis of HSP as the cause for severe disfiguring scalp and facial oedema and hypertensive encephalopathy was made based on the characteristic purpuric rash, arthritis, and abdominal pain. He was commenced on intravenous methylprednisolone 0.5 mg/kg 6-hourly which was converted to oral prednisolone 1 mg/kg/day after 3 days. The blood pressure was gradually lowered using intravenous hydralazine infusion and later with oral nifedipine and prazosin. Following treatment, the scalp and facial oedema rapidly improved (Figures 1(b)–1(d)); however, his blood pressure remained high (between 95^th^–99^th^ centiles). His urine analysis which was repeatedly normal during the first two weeks showed 10–12 red blood cells/hpf on the 14^th^ day of illness. The renal biopsy that was performed due to the severity of presentation and persistent hypertension revealed normal light microscopical appearance of glomeruli, tubulointerstitium, and blood vessels without overt signs of leukocytoclastic vasculitis. However, the strong IgA granular staining (3+) and moderate IgG granular staining (2+) in the mesangium in immunofluorescence suggested the diagnosis of HSP nephritis.

## 3. Discussion

HSP is a leukocytoclastic vasculitis that involves small blood vessels [1]. It occurs due to the deposition of IgA_1_, a subclass of IgA, in the vessel walls of the skin, gastrointestinal tract, kidney, and joints. The diagnosis of HSP is based on several criteria. According to the most recent consensus criteria published in 2010 by the European League Against Rheumatism (EULAR), Paediatric Rheumatology International Trials Organisation (PRINTO), and Paediatric Rheumatology European Society (PRES), the diagnosis of HSP is confirmed by demonstrating the presence of nonthrombocytopenic palpable purpura with at least one of the following: diffuse abdominal pain, typical vasculitis or proliferative glomerulonephritis with predominant IgA deposition on the skin or renal biopsy specimens, arthritis or arthralgia, or renal involvement indicated by haematuria or proteinuria [1, 2]. The patient described in this case report had all these features, confirming the diagnosis of HSP.

The most salient feature of this case report is the presence of severe disfiguring oedema involving the face and the scalp that was awfully painful in this boy. Although subcutaneous oedema is commonly reported in HSP, it occurs in the dependent areas of the body [1]. Face or scalp oedema is uncommon especially in children older than 2 years. The few reported patients had mild-to-moderate oedema of the face; however, massive disfiguring oedema of this nature is extremely rare [3–6]. Subcutaneous oedema in HSP is likely to be due to the inflammation of vessels which leads to fluid leakage but hypertension causing increased intravascular pressure may also contribute.

Other atypical cutaneous manifestations of HSP already reported in the literature include Köbnerization, Rumpel–Leede capillary fragility phenomenon, and blistering eruptions [7–9]. Köbnerization refers to appearance of nonblanching palpable skin lesions following mechanical friction, whereas the Rumpel–Leede capillary fragility phenomenon is the appearance of petechiae in an extremity following the application of a tourniquet [7]. A recent systematic review reported that <2% of patients with HSP develop blistering skin lesions. Of the 41 patients described in this systematic review, both purpura and blisters showed similar distribution and appeared either concomitantly or with a latency of up to 14 days [9].

Another unusual feature observed in this child is severe hypertension and hypertensive encephalopathy in the absence of clinical features of overt nephropathy. Headache, visual disturbances, intractable vomiting, exaggerated tendon reflexes, and extensor plantar responses in our child were suggestive of hypertensive encephalopathy or posterior reversible encephalopathy syndrome (PRES) [10]. Noncontrast CT brain of this child did not show features of PRES; however, MRI brain would have been more informative to diagnose PRES.

The treatment of HSP is mainly supportive. Although steroids are widely used in the treatment, the existing evidence show them to be effective only for abdominal pain associated with HSP. Specifically, steroids are not effective for cutaneous manifestations [7]. Furthermore, unnecessary treatment with steroids may result in worsening of hypertension which could complicate the disease course.

In conclusion, this case report presents a child with severe, disfiguring scalp and facial oedema due to HSP. It highlights that severe subcutaneous oedema of HSP can involve any part of the body not limiting to dependent areas. This case also highlights that severe hypertension could occur in a child with HSP even without overt nephritis or renal dysfunction.

## Data Availability

Clinical data of the patient were gathered while managing the child and rechecked from the bed head tickets and personal clinic records of the child. These data used to support the findings of this case report are included within the article. Consent was taken from parents for the publication of the child's details and photographs, not for sharing the copies of the bed head tickets and personal clinic records of the child. Scanned documents of the personal clinic records can be provided on request after getting consent from the patient's parents and permission from the director of hospital. Scanned documents of bed head tickets cannot be provided as it is not ethical and illegal in Sri Lanka.
